# Genetic Analysis of Genomic and Methylomic Variation and Identification of Multi-Trait Mutants in Rice Carried on Chang’e-5

**DOI:** 10.3390/plants15172614

**Published:** 2026-08-27

**Authors:** Kai Sun, Jinrui Li, Zhe Zhao, Shiyi Chen, Yuanyuan Guo, Ying Ling, Yongfen Wang, Zhikai Han, Zengtong Luo, Wuming Xiao, Jiafeng Wang, Guili Yang, Yongzhu Liu, Tao Guo, Chun Chen

**Affiliations:** 1 National Engineering Research Center of Plant Space Breeding, South China Agricultural University, Guangzhou 510642, China; 2 College of Agriculture, South China Agricultural University, Guangzhou 510642, China; 3 Guangdong Basic Research Center of Excellence for Precise Breeding of Future Crops, South China Agricultural University, Guangzhou 510642, China; 4 Guangdong Zhaohua Aerospace Breeding Innovation Research Institute, Heyuan 517000, China

**Keywords:** rice, space mutagenesis, genomic variation, DNA methylation, mutant identification

## Abstract

Global food security is facing challenges from population growth to diminishing arable land. Space mutation breeding holds promise for overcoming the variation limitations in conventional breeding; however, the mutagenic effects of the deep-space environment on rice and the transgenerational inheritance patterns of induced variations remain unclear. In this study, rice seeds carried by the Chang’e-5 spacecraft were used as materials. Whole-genome sequencing and whole-genome bisulfite sequencing were performed on the first (SP_1_) and second generations (SP_2_) of space-mutagenized plants after their return to Earth. The results showed that the number of genomic variants in the SP_2_ generation increased significantly compared with SP_1_, and SNPs, homozygous sites, and variants in coding regions were more heritable. The genome-wide methylation level was elevated in the SP_2_ generation, and among differentially methylated cytosines, those in the CG context exhibited the highest heritability. Furthermore, large-scale screening for nitrogen efficiency, tolerance to PEG-induced stress, and germination-stage cold resistant mutants was conducted in the SP_2_ generation, and phenotypic validation was performed in the third generation (SP_3_). By integrating multi-omics analyses of representative mutants to mine candidate genes, a number of heritable elite mutants were obtained, and seven candidate genes for key traits were identified. This study systematically elucidates the transgenerational inheritance patterns of deep-space-induced variation in rice. The multi-trait mutants obtained provide valuable germplasm resources for gene cloning and breeding applications in rice.

## 1. Introduction

With the continuous growth of global population, the increasing scarcity of arable land resources, and the uncertainty brought by climate change to agricultural production, food security has become one of the major challenges facing humanity in the 21st century [1,2]. Rice is the staple food for over half of the world’s population, and its stable yield and improved quality are crucial for ensuring food security [3]. Traditional hybridization and mutagenesis breeding techniques have made significant contributions to the genetic improvement of rice, but their variation range is limited and their cycle is long, making it difficult to meet the urgent demand for breakthrough new germplasm. Space mutagenesis breeding, as an emerging technology that utilizes special space environments to induce genetic variation in organisms, has opened up a new path for crop genetic improvement.

When organisms are exposed to the space environment, they encounter extreme conditions that are completely different from the ground. This environment mainly includes space radiation, microgravity, extreme temperature, and magnetic field changes, all of which together constitute a unique mutagenic source [4,5]. Compared with common ground induced mutations such as gamma rays and ion beams, space radiation has characteristics of high linear energy transfer (LET), strong biological effects, and complex damage repair, and is believed to induce more diverse and rare genetic variations [6,7,8]. When organisms face the extreme space environment mentioned above, they will generate sustained stress responses, causing DNA damage and epigenetic modifications, ultimately leading to a series of unique mutation spectra at the genome sequence and epigenome levels that are different from ground mutagenesis. These mutations are also the genetic basis for the emergence of new traits [9,10].

In recent decades, humans have screened batches of new germplasm with high yield, high quality, and disease tolerance in various crops such as rice, wheat, and vegetables through space mutagenesis technology, which fully demonstrates the potential application of space mutagenesis breeding [11,12,13]. However, compared to the achievements made in practical applications, our understanding of the fundamental genetic laws behind space mutagenesis is still quite limited. Most existing research remains at the level of phenotype observation and preliminary molecular marker identification, lacking a systematic and high-precision analysis of the types of genomic variations in organisms after space flight [14,15]. At the same time, whether the mutations generated by space mutagenesis can be stably inherited is the key to breeding applications. However, existing research mostly focuses on observing a certain generation, and little is known about the rules of mutation transmission across generations [16,17]. The shortcomings of these studies greatly constrain the leap of space mutagenesis technology from “experience screening” to “precise design”.

At the trait-mining level, existing space mutagenesis studies have largely focused on screening for a single or a few agronomic traits, failing to fully unlock the abundant genetic diversity harbored in mutagenized populations and lacking systematic, large-scale characterization of key breeding traits such as nutrient efficiency and abiotic stress tolerance [18]. Moreover, most studies have not linked population-level genetic variation patterns to the molecular mechanisms underlying specific traits, making it difficult to establish a complete chain from mutagenic effects through genetic variation to phenotypic changes, which hinders precise functional gene mining in subsequent analyses [19].

**Figure 1:**
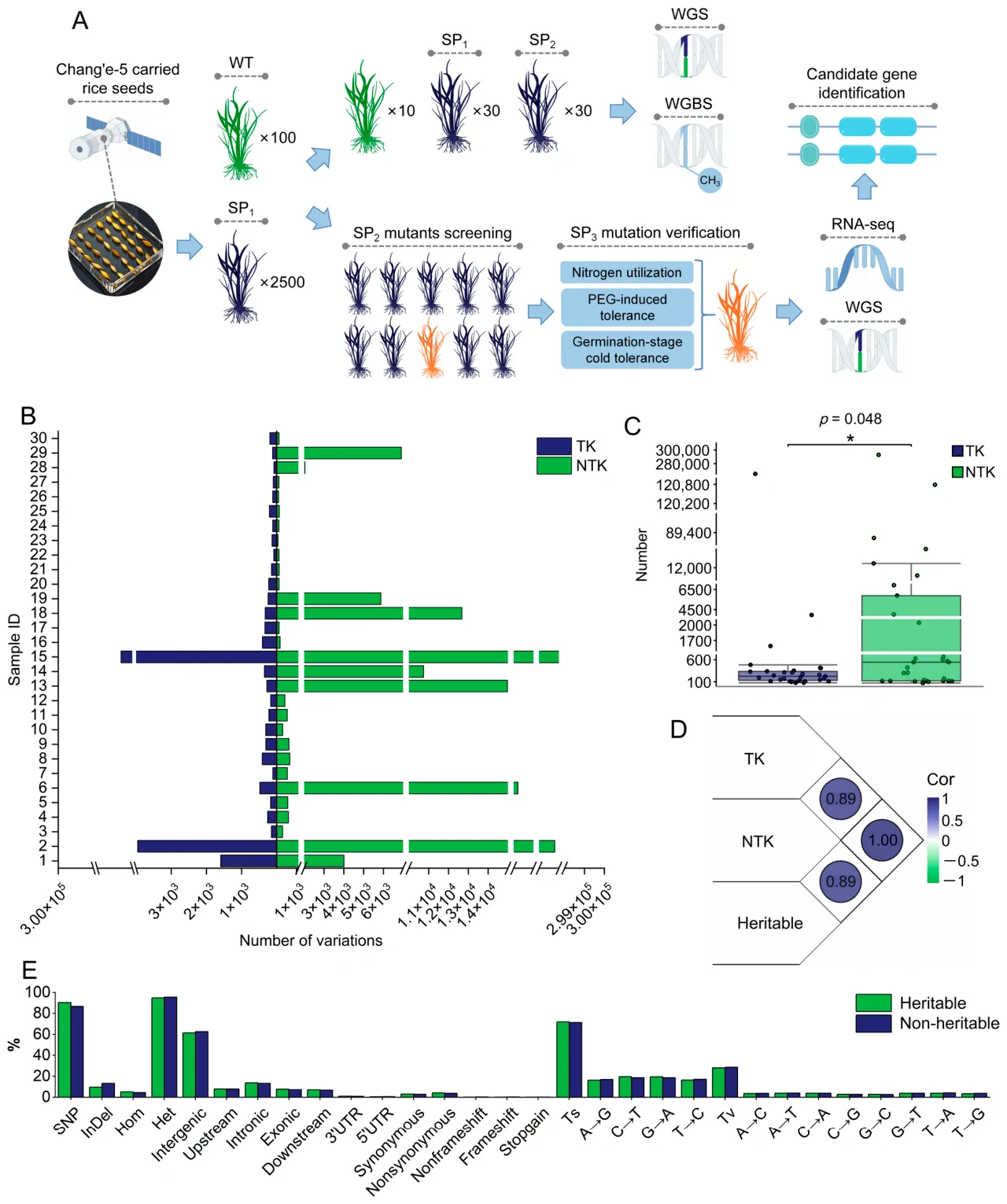
Study design and genetic analysis of genomic variations. (**A**) Research plan design. (**B**) The number of genomic variations in individual samples of TK and NTK. (**C**) Comparison of the number of genomic variations between TK and NTK. The *y*-axis is broken to accommodate the wide range of variation in mutation counts among individual samples. * *p* < 0.05. (**D**) The correlation between the total variability of TK and NTK and the number of heritable loci. (**E**) Comparison of genomic variation characteristics between heritable loci and non-heritable loci. (**C**) was analyzed using an independent-sample two-tailed *t*-test. The *p* = 0.048 was obtained from a paired *t*-test on the total number of variants in the 30 paired TK-NTK samples. (**D**) was analyzed using Pearson correlation coefficient, calculated based on the log10-transformed number of variant sites (log10 transformation was applied to make the data more normally distributed).

As an important platform for deep-space exploration, the Chang’e-5 mission carried seeds that were exposed to the lunar orbital environment, where the composition and dose of space radiation differ from those encountered in low Earth orbit missions, thus possessing the potential to induce unique variations. This study conducted high-depth whole-genome sequencing (WGS) and whole-genome bisulfite sequencing (WGBS) on the space mutagenesis first generation (SP_1_) and space mutagenesis second generation (SP_2_) of rice seeds carried on Chang’e-5 after returning to the ground, identifying space environment induced genomic and methylomic variations, and systematically elucidating their genetic and evolutionary patterns across different generations; large-scale and multi-trait mutant phenotype identification was conducted on SP_2_, and it was validated in the space mutagenesis third generation (SP_3_) to comprehensively explore the phenotypic diversity generated by space mutagenesis. By integrating whole-genome variant information from representative mutants with multi-time-point RNA sequencing (RNA-seq) analysis, we revealed the dynamic molecular response network underlying trait formation and identified candidate genes regulating key traits (Figure 1A), and their expression levels were validated by quantitative reverse transcription polymerase chain reaction (qRT-PCR). The results of this study will systematically reveal the mutagenic effects of deep-space environment on rice and its transgenerational inheritance patterns. The identified mutants provide direct support for the cloning of genes underlying important traits and the development of novel breeding materials.

## 2. Results

### 2.1. Analysis of Genetic Patterns of Genomic Variation

To clarify the genetic patterns of genomic variations caused by deep-space flight across different generations, WGS was conducted separately for TK and NTK. In total, 30 TK and 10 CK obtained 392.6 Gb of clean data through sequencing, with an average sequencing depth of 25.3×. After aligning with the reference genome SK1 (the background reference genome of the HJXSM variety previously constructed by our research group) [20], a total of 276,898 authentic mutation sites were screened. The mutation frequency of the samples ranged from 1.96 × 10^−7^ to 6.08 × 10^−4^ variants per base pair sequenced, and there were significant differences in the number of mutation sites between different samples. Among them, TK15 had the highest number of mutation sites, reaching 265,320, while TK28 had the lowest number of mutation sites, at only 78 (Figure 1B). A total of 251.9 Gb of clean data was obtained from 30 NTKs in SP_2_, with an average sequencing depth of 21.7×. A total of 565,698 authentic mutation sites were screened, with sample mutation frequencies ranging from 1.84 × 10^−7^ to 7.38 × 10^−4^ variants per base pair sequenced. Among them, NTK15 had the highest number of mutation sites (292,608), while NTK23 had the lowest number (73). These loci were evenly distributed on the chromosomes and no obvious hotspot areas were found (Figure S1). Compared with TK, the number of genomic variations in NTK significantly increased (Figure 1C). A comparison of the genomic variation characteristics between TK and NTK revealed that the proportion of single nucleotide polymorphisms (SNPs), synonymous single nucleotide variant (SNV), nonsynonymous SNV, and transitions in NTK significantly increased, while the proportion of InDel, 5′UTR, frameshift, nonframeshift, and transversions significantly decreased (Figure S2A).

Further, the genomic variations in TK and NTK were intersected, with the intersection being the genetic locus and the rest being non-heritable loci. In total, 40.75% of the mutation sites in TK are inherited from NTK, with a total of 112,837. The number of heritable loci in the 30 samples ranged from 0 to 112,040, with the TK15-NTK15 group having the highest proportion of genetic loci, accounting for 42.25% of the total number of variant sites in TK15, while the TK25-NTK25 group had no genetic mutation sites (Figure S2B). A significant positive correlation was observed between the total variation in TK and the total variation in and heritable loci of NTK, that is, the greater the total variation in TK, the more heritable loci and total variation in NTK (Figure 1D). The variation characteristics in heritable and non-heritable loci were compared, and it was found that the proportion of SNP, homozygous sites, coding sequence (CDS) region, synonymous SNV, nonsynonymous SNV, and C/G → A/T increased in heritable loci, while the proportion of InDel, heterozygous, intergenic, frameshift, nonframeshift, and A/T → C/G decreased (Figure 1E). In addition, enrichment analysis was conducted on genes containing heritable loci and non-heritable loci. The results showed that genes containing heritable loci were mainly involved in various biological processes such as DNA activity, nucleotide metabolism, and protein modification, and were related to homologous recombination, sulfur transfer systems, pyrimidine metabolism, and the synthesis and metabolic pathways of various organic compounds; genes containing non-heritable sites were mainly involved in various DNA dynamic changes, as well as RNA synthesis and metabolism processes. They are also related to homologous recombination, plant hormone signal transduction, and the synthesis and metabolism of various organic compounds (Figure S3A,B).

In summary, the number of genomic variations in NTK was significantly increased compared to TK, with SNP, homozygous, CDS regions, synonymous SNV, nonsynonymous SNV, as well as mutation sites on genes related to nucleotide metabolism, protein modification, sulfur transfer system, pyrimidine metabolism, and other functions being more easily inherited.

### 2.2. Analysis of Genetic Patterns of DNA Methylation

**Figure 2:**
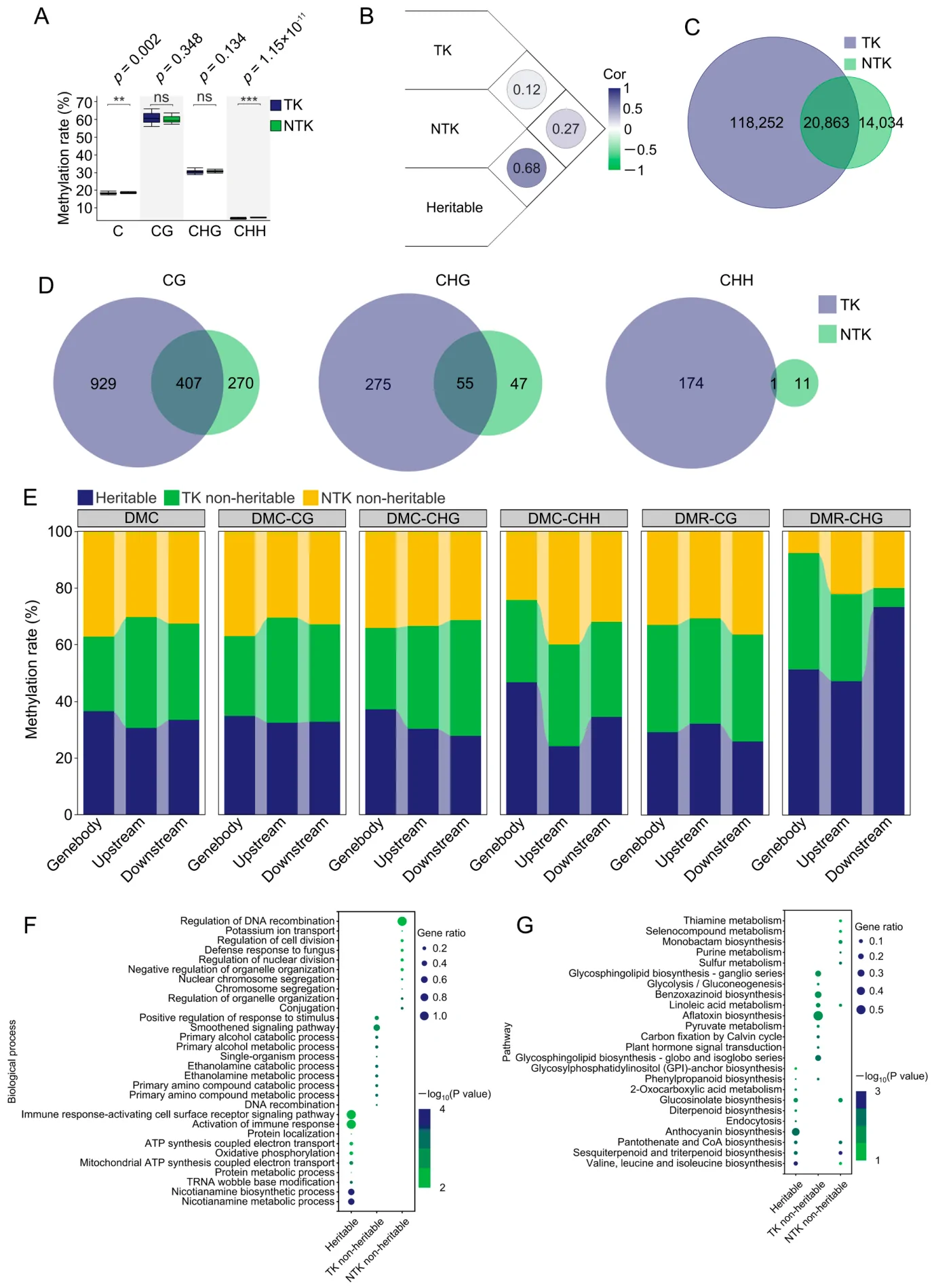
The genetic patterns of DNA methylation in TK and NTK. (**A**) Comparison of methylation rates between TK and NTK types. ns *p* ≥ 0.05, ** *p* < 0.01, *** *p* < 0.001. (**B**) The correlation between TK, NTK, and heritable methylation sites. (**C**) Heritable differentially methylated cytosines (DMCs) and non-heritable DMCs of TK and NTK. (**D**) The heritable and non-heritable quantities of three types of differentially methylated regions (DMRs). (**E**) The proportion of different types of heritable and non-heritable DMCs and DMRs in the genome. (**F**) Gene Ontology (GO) enrichment of heritable and non-heritable DMRs genes, with the top 10 biological processes of significance. (**G**) Kyoto Encyclopedia of Genes and Genomes (KEGG) enrichment of heritable and non-heritable DMRs genes, with the top 10 pathways of significance. (**A**) was analyzed using Duncan’s multiple range test. (**B**) was analyzed using Pearson correlation coefficient, based on the log10-transformed number of DMCs.

To clarify the genetic patterns of DNA methylation induced by deep-space flight across different generations, WGBS was conducted separately for TK and NTK. Compared with TK, the overall methylation rate of NTK was increased, especially the methylation rate of CHH type which was significantly increased (Figure 2A). The methylation rates of TK and NTK at different gene positions were compared, and it was found that the methylation rate of NTK was significantly increased in the upstream, intron, and 3′UTR of the gene. From the perspective of different methylation types, the methylation rate of CHH in the entire NTK genome was significantly increased, while the methylation rate of CHG was only significantly increased in introns and 3′UTR, whereas the methylation rate of CG was decreased in 3′UTR (Figure S4). In addition, in contrast to genomic variations, no significant correlation was observed between TK, NTK, and genetic methylation sites (Figure 2B).

Further screening was conducted to identify differentially methyla ted cytosines (DMCs) and differentially methylated regions (DMRs) of TK and NTK. A total of 20,863 DMCs were inherited from TK to NTK, accounting for 15% of the total DMCs in TK and 59.78% of the total DMCs in NTK, respectively (Figure 2C). The distribution of heritable and non-heritable DMCs on chromosomes is shown in Figure S5A. At the same time, a total of 463 DMRs were inherited from TK to NTK, and their distribution on chromosomes is shown in Figure S5B–D. Among them, the CG type had the highest number of DMRs, followed by CHG, while the CHH type had only one DMR (Figure 2D). The distribution of heritable and non-heritable DMCs and DMRs on the genome was compared, and the results showed that all three types of heritable DMCs had a higher proportion in the gene body, while non-heritable DMCs had a higher proportion upstream and downstream of the gene; conversely, DMRs had a higher proportion both upstream and downstream of the gene, while their proportion in the gene itself was lower (Figure 2E). Enrichment analysis was conducted on genes containing heritable DMRs and genes containing non-heritable DMRs. Gene Ontology (GO) enrichment results showed that heritable DMRs genes were mainly involved in biological processes such as tobacco amine synthesis and metabolism, protein activity, oxidative phosphorylation, immune response, and ATP electron transfer; non-heritable DMRs genes were mainly involved in DNA and chromosome activity, response to stimuli, cell division, and metabolic pathways of various organic compounds (Figure 2F). The Kyoto Encyclopedia of Genes and Genomes (KEGG) enrichment results showed that heritable DMRs genes were mainly involved in the synthesis and metabolism of various organic compounds such as anthocyanins and diterpenes, while non-heritable DMRs genes were mainly involved in plant hormone signal transduction, Calvin cycle, glycolysis, synthesis and metabolism of inorganic compounds such as sulfur, and synthesis and metabolism pathways of organic compounds such as phenylpropanoid (Figure 2G).

In summary, similar to the genetic pattern of genomic variation, the genome-wide methylation rate of NTK is significantly higher than that of TK. CG had the highest proportion of heritable methylation sites, and DMCs on the gene body, DMRs on the upstream and downstream of the gene, as well as DMRs on genes related to grass amine synthesis and metabolism, protein activity, oxidative phosphorylation, immune response, ATP electron transfer, anthocyanins, diterpenes and other organic compounds were more easily inherited.

### 2.3. Screening of Multi-Trait Mutants

The above results elucidate the transgenerational inheritance patterns of space mutagenesis induced variation and reveal that the SP_2_ generation accumulates a large number of heritable functional variants, which are enriched in stress response, nutrient metabolism, and basic biosynthetic pathways. This suggests that the Chang’e-5 mutagenized population harbors abundant trait diversity, with considerable potential for mining, particularly in abiotic stress tolerance and nutrient efficiency. High nitrogen use efficiency, tolerance to PEG-induced stress, and germination-stage cold tolerance are key traits that constrain rice yield and quality and represent important targets in current rice genetic improvement; their molecular regulatory networks align with the heritable variation enrichment pathways observed in this study.

**Figure 3:**
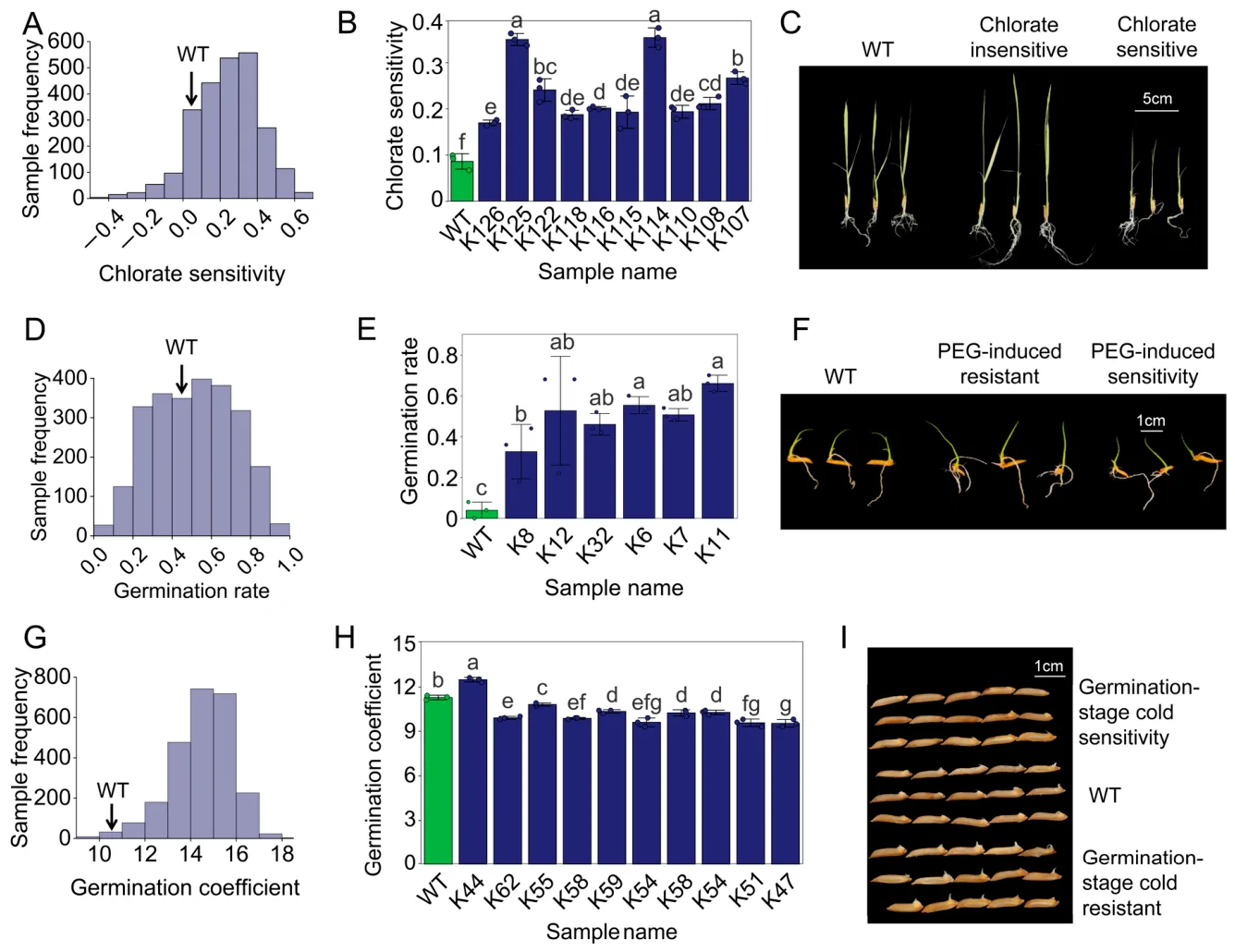
Screening of multi-trait mutants. (**A**) Frequency distribution of chlorate sensitivity in space mutation second generation (SP_2_) mutant. (**B**) Space mutation third generation (SP_3_) partial nitrogen utilization mutation validation. (**C**) Comparison of chlorate-insensitive mutants, chlorate-sensitive mutants, and the wild type (WT). (**D**) Frequency distribution of germination rate of SP_2_ mutant after PEG-induced drought treatment. (**E**) SP_3_ partial PEG-induced tolerant mutation validation. (**F**) Comparison of PEG-induced tolerant mutants, PEG-induced sensitivity mutants, and WT. (**G**) Frequency distribution of germination coefficient of SP_2_ mutant after germination-stage cold treatment. (**H**) SP_3_ partial germination-stage cold-tolerant mutation validation. (**I**) Comparison of germination-stage cold resistant mutants, cold sensitivity mutants, and WT. Data are shown as mean ± SD (n = 3) in (**B**,**E**,**H**), different lowercase letters above the bars indicate significant differences at *p* < 0.05 according to Duncan’s multiple range test.

In this study, a chlorate screening system was used for mutant selection. The culture medium was a nutrient solution containing KNO_3_ as the sole nitrogen source. Chlorate, a structural analog of nitrate, is taken up by nitrate transporters and subsequently reduced by nitrate reductase to toxic chlorite, causing cellular damage. Under nitrogen-limited conditions (with KNO_3_ as the sole nitrogen source), chlorate-sensitive materials generally possess higher nitrate uptake and reduction capacities, which can be converted into higher nitrogen use efficiency when nitrogen supply is sufficient [21,22,23]. Screening and identification of mutants were performed for 2500 lines (25,000 individual plants) of SP_2_. Statistical analysis was conducted on the chlorate sensitivity of different materials, and among 2500 lines, the chlorate sensitivity ranged from −46.74% to 69.29% (Figure 3A). A total of 88 materials with the highest and lowest chlorate sensitivity were retained, and their chlorate inhibition rates were further identified in SP_3_ (Figure 3B). Materials with consistent changes in representative types from two generations were retained. Finally, 52 mutants were screened, including 32 nitrogen efficient mutants and 20 chlorate insensitive mutants (Figure 3C).

After PEG-induced drought treatment, the germination rate of SP_2_ seeds ranged from 1.03% to 98% (Figure 3D). A total of 79 materials with the highest germination rate were preliminarily selected for subsequent validation. After drought tolerance testing of SP_3_ (Figure 3E), a total of 32 drought-tolerant mutants were screened (Figure 3F).

The germination coefficient of SP_2_ after cold treatment was between 9.09 and 18.08 (Figure 3G). The germination coefficient of germination-stage cold-tolerant materials is high, usually starting from the 5th day, and the bud length is longer, with a final germination rate greater than 80%; in contrast, the germination coefficient of germination-stage cold sensitive materials is low, with germination starting from the 7th day and shorter bud length, resulting in a final germination rate of less than 50%. Based on the germination coefficient, 41 germination-stage cold-tolerant mutants were preliminarily screened in SP_2_, and after verification of the germination-stage cold-tolerant phenotype in SP_3_ (Figure 3H), a total of 27 germination-stage cold-tolerant mutants were obtained (Figure 3I).

### 2.4. Genomic Variation Characteristics of Representative Mutants

**Figure 4:**
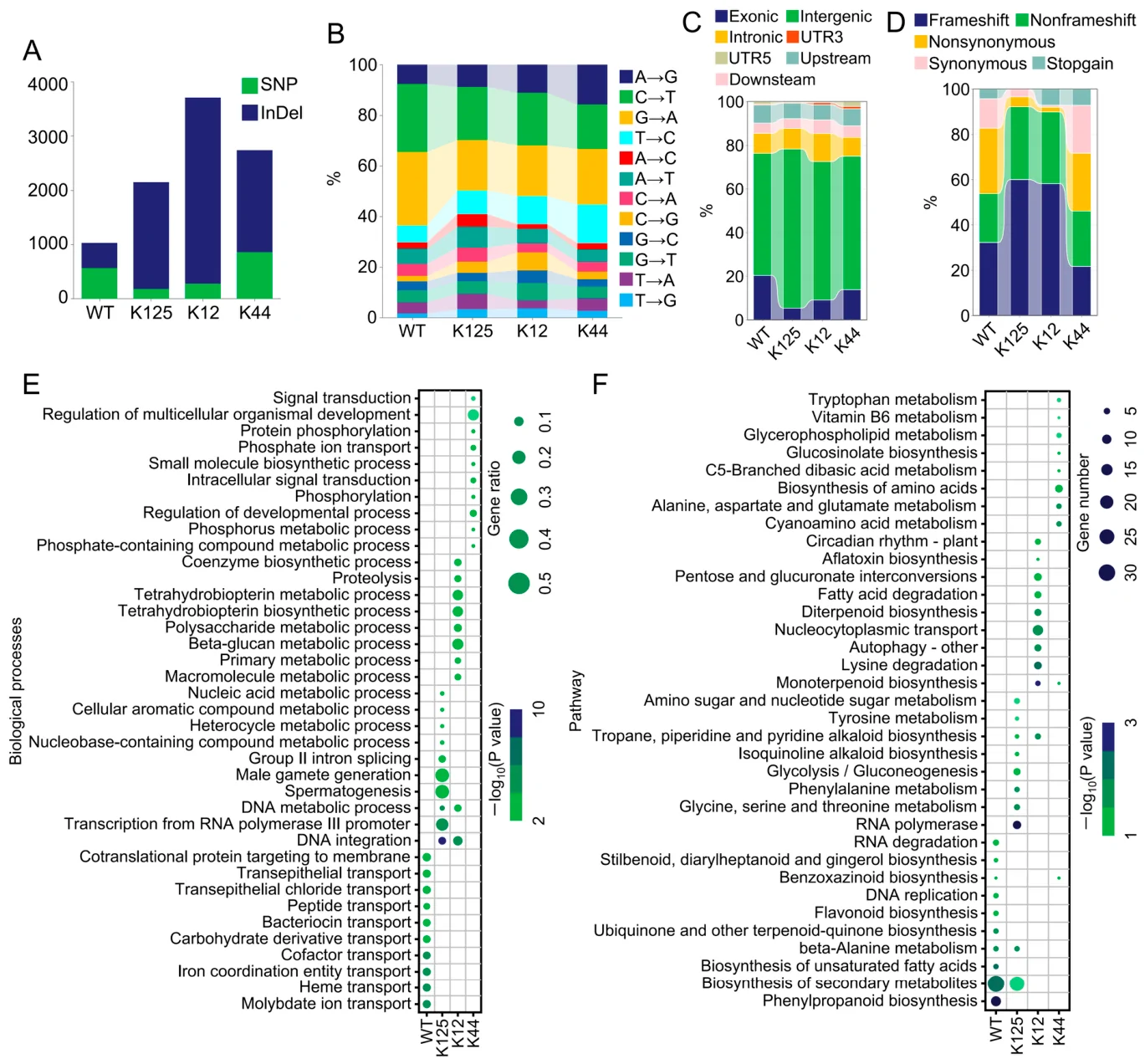
Genomic variation characteristics in the 3 representative mutants. (**A**) Comparison of the number and types of genomic variants among the 3 mutants and the wild type (WT). (**B**) Comparison of base transitions and transversions among the 3 mutants and the WT. (**C**) Comparison of the genomic distribution characteristics of variant sites between WT and the three mutants. (**D**) Comparison of the proportions of functional variant types among variant sites between WT and the three mutants. (**E**) Comparison of Gene Ontology (GO) enrichment of genes carrying variants among the 3 mutants and the WT. (**F**) Comparison of Kyoto Encyclopedia of Genes and Genomes (KEGG) enrichment of genes carrying variants among the 3 mutants and the WT.

To characterize the genomic variation profiles of the mutants, one representative mutant exhibiting the most pronounced phenotypic difference was selected from each of the nitrogen efficient, PEG-induced tolerant, and germination-stage cold-tolerant mutant groups (K125, K12, and K44, respectively) for WGS, with WT control included (Figure S6A–C). After filtering out background false-positive sites, the total numbers of variant sites in K125, K12, and K44 reached 2149, 3707, and 2778, respectively, representing a 2- to 4-fold increase compared with WT (Figure 4A). Unlike WT, all three mutants showed a greater increase in the number of InDels than in SNPs. Comparison of base substitution patterns of SNPs revealed that the proportion of transitions decreased in all three mutants, whereas the proportion of transversions increased to varying degrees relative to WT (Figure 4B). Analysis of the genomic distribution of variants across functional regions showed that in both WT and the three mutants, variants were predominantly located in intergenic regions, accounting for more than 60% of the total (Figure 4C,D). Compared with WT, the three mutants exhibited a reduced proportion of variants in coding regions. Further functional effect annotation of coding region variants revealed that the proportions of high impact functional mutations (nonsynonymous mutations, frameshift mutations, and stopgain mutations) increased to varying degrees.

To further dissect the functional preferences of the mutated genes, GO and KEGG enrichment analyses were performed on the gene sets carrying variants. GO enrichment results showed that, compared with WT, mutated genes in K125 were most significantly enriched in processes such as DNA activity, nucleic acid metabolism, and gametogenesis, mutated genes in the PEG-induced tolerant mutant K12 were more enriched in pathways such as DNA metabolism and polysaccharide metabolism, and mutated genes in the germination-stage cold-tolerant mutant K44 were significantly enriched in processes including phosphorus metabolism, phosphorylation, and signal transduction (Figure 4E). KEGG pathway enrichment analysis revealed that the mutated genes from all three mutants were commonly enriched in basic metabolic pathways such as phenylpropanoid biosynthesis, biosynthesis of secondary metabolites, and biosynthesis of amino acids, while also showing specific enrichment in stress-related pathways such as plant hormone signal transduction and circadian rhythm regulation in each mutant (Figure 4F).

### 2.5. RNA-Seq of Representative Mutants

**Figure 5:**
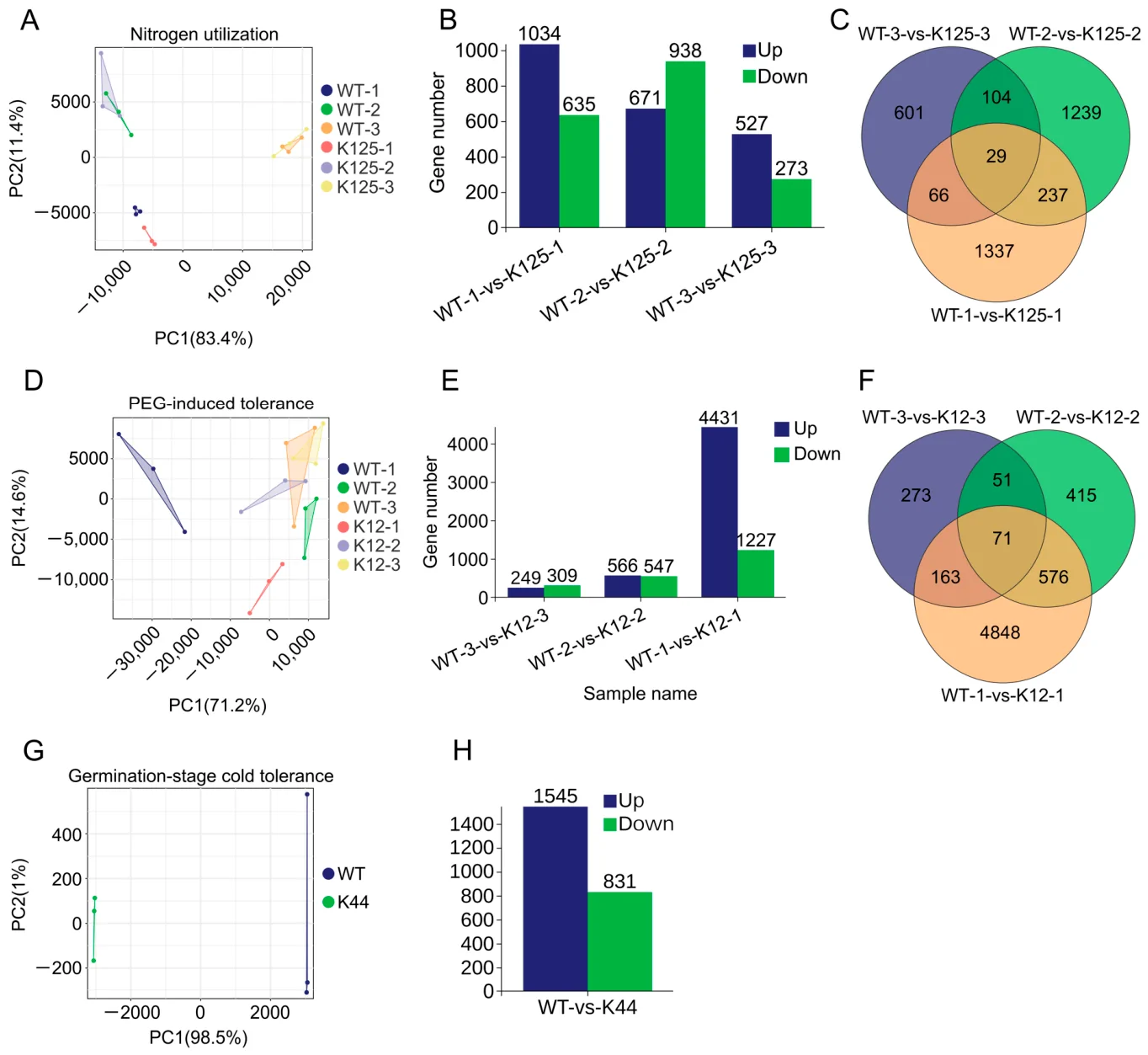
RNA sequencing (RNA-seq) representing mutants. (**A**) Principal component analysis (PCA) plot of K125. (**B**) Differentially expressed genes (DEGs) at different sampling times for K125. (**C**) Intersection of DEGs at different sampling times for K125. (**D**) PCA plot of K12. (**E**) DEGs at different sampling times for K12. (**F**) Intersection of DEGs at different sampling times for K12. (**G**) PCA plot of K44. (**H**) DEGs at different sampling times for K44.

RNA-seq was performed on the three mutants sampled under different treatments and at multiple time points. K125 and WT showed clustering within sample groups treated differently, while exhibiting discrete distributions between groups (Figure 5A), indicating that different treatments significantly affected gene expression patterns. A total of 3613 differentially expressed genes (DEGs) were screened, and the number of DEGs gradually decreased with the delay of sampling time (Figure 5B). Among them, 29 genes were differentially expressed in all three treatments (Figure 5C). GO and KEGG enrichment analyses were performed on them separately, and the results showed that on the 8th day, the functions of DEGs were mainly related to DNA activity, cell cycle, and the synthesis and metabolism of organic compounds such as peptides and flavonoids. After KNO_3_ treatment on the 12th day, the main functions of DEGs shifted to cell and component movement, stimulus response, hormone and signal transduction, etc. After treatment with KClO_3_ on the 12th day, the main function of DEGs was mainly related to oxidative stress. Fatty acid and phenylpropanoid biosynthesis played a role throughout the entire sampling period (Figure S7A,B). The functional center transformation of the above DEGs after different treatments also reflects the mutant’s process of nitrogen utilization.

PEG-induced treatment significantly affected the gene expression patterns of K12 (Figure 5D). After screening, a total of 6361 DEGs were obtained, with the highest number of DEGs at the 3rd day of PEG-induced treatment, while the number of DEGs at the 5th and the 7th day decreased significantly (Figure 5E), indicating that the seed germination response was more pronounced in the early stages of PEG-induced treatment, with 71 genes being differentially expressed in all three treatments (Figure 5F). The GO and KEGG enrichment results indicate that the function of DEGs during PEG-induced treatment for the 3rd day is mostly related to oxidative stress and carbohydrate synthesis metabolism; the function of DEGs at the 5th day of PEG-induced treatment is similar to that at the 3rd day, and is related not only to oxidative stress but also to stimulus response and detoxification response; after the 7th day of PEG-induced treatment, the function of DEGs shifted towards conventional life activities such as the synthesis and metabolism of various organic compounds (Figure S8A,B). The above results indicate that the early stage of seed germination is the main period for responding to PEG-induced stress, and oxidative stress may be an important pathway for seeds to respond to PEG-induced stress.

A total of 2376 differentially expressed genes (DEGs) were identified in K44, among which the number of upregulated DEGs was higher than that of downregulated DEGs (Figure 5G,H). Enrichment analysis was conducted on them, and DEGs were found to be related to the synthesis and metabolism of various organic compounds such as oxidation–reduction and sugars, as well as the synthesis of substances such as phenylpropanoid (Figure S9A,B).

The expression levels of known marker genes related to nitrogen utilization, drought, and cold stress responses in each mutant were compared with those in the WT. The results showed that multiple marker genes exhibited significant differences in expression, confirming the reliability of the RNA-seq data (Figure S10).

### 2.6. Identification and Validation of Candidate Genes

**Figure 6:**
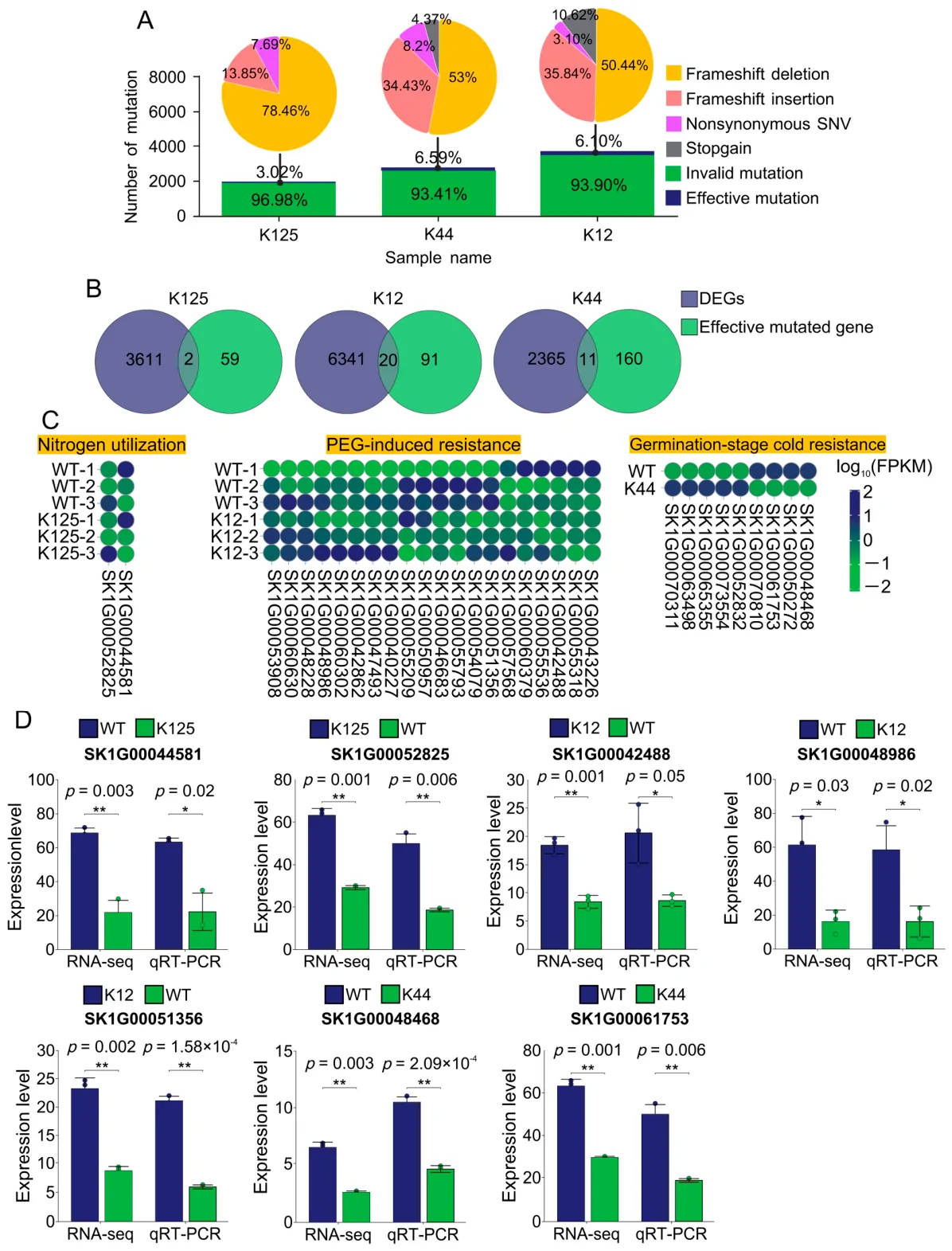
Identification of candidate genes. (**A**) The proportion of high impact mutations among the 3 mutant genomes, as well as the proportion of various types of functional mutations. (**B**) The intersection of RNA sequencing (RNA-seq) differentially expressed genes (DEGs) and genes containing high impact variants. (**C**) The expression heatmap of potential candidate genes obtained through preliminary screening. (**D**) Quantitative reverse transcription polymerase chain reaction (qRT-PCR) validation of 7 candidate genes. * *p* < 0.05, ** *p* < 0.01.

To further identify candidate genes responsible for the phenotypic changes in the mutants, the genomic variation data and RNA-seq results were jointly analyzed. According to the results of the mutant library, K125 had a total of 2149 mutation sites and 65 high impact mutation sites, distributed across 61 genes, of which 92.31% were frameshift mutations (Figure 6A). An intersection of the 61 mutated genes with the 3613 DEGs resulted in two genes (Figure 6B), namely *SK1G00044581* and *SK1G00052825*. In the K125 mutant, *SK1G00044581* has a 7 bp frameshift deletion in the 11th exon region, resulting in a significant decrease in its expression level after KNO_3_ treatment. *SK1G00052825* has a 2 bp frameshift deletion in the first exon, and its expression level increased significantly after KClO_3_ treatment, reflecting the difference in response mechanisms between the two.

K12 had a total of 3707 mutation sites and 226 high impact mutation sites, distributed across 211 genes, with frameshift mutations accounting for 87.43% (Figure 6A). Taking the intersection of the 211 mutated genes and the 6361 DEGs, a total of 20 genes were obtained (Figure 6B). Further analysis of the functions and pathways of these 20 genes, combined with existing reports, was focused on oxidative stress, ion transport, and other factors related to PEG-induced tolerance. Finally, three genes were selected, namely *SK1G00042488*, *SK1G00048986*, and *SK1G00051356*. There is a 4 bp frameshift insertion in the third exon of *SK1G00042488*, resulting in a significant decrease in its expression level on the 3rd day of PEG-induced treatment. There is a 1 bp frameshift insertion in the first exon of *SK1G00048986*, which also leads to a significant decrease in its expression level on the 3rd day of PEG-induced treatment. There is a 20 bp frameshift deletion in the first exon of *SK1G00051356*, resulting in a significant increase in its expression level on the 3rd day of PEG-induced treatment. Although the three candidate genes have different modes of action, their expression levels changed significantly on the third day, indicating the importance of early response to seed germination after PEG-induced treatment.

K44 had a total of 2778 mutation sites and 183 high impact mutation sites, distributed across 171 genes, with frameshift mutations accounting for 93.69% (Figure 6A). Taking the intersection of the 171 mutated genes and the 2376 DEGs, a total of 11 genes were obtained. Further screening based on gene function and previous reports resulted in two candidate genes (Figure 6B), *SK1G00061753* and *SK1G00048468*. *SK1G00061753* has a 2 bp frameshift insertion in the second exon, and *SK1G00048468* has an 88 bp frameshift insertion in the sixth exon, both of which result in a significant decrease in gene expression.

In summary, based on the variation information of the mutant and the DEGs of the transcriptome, 33 potential candidate genes were preliminarily screened, and their expression levels are shown in Figure 6C. Based on the functions and pathways of the genes, combined with existing reports, seven candidate genes were ultimately screened (Table 1). To verify the authenticity of the seven candidate genes mentioned above, their expression levels were detected by qRT-PCR. The results showed that the expression levels of the seven candidate genes were consistent with the trend of transcriptome results (Figure 6D), and multiple genes had been reported by previous researchers, proving the authenticity and reliability of the candidate genes selected in this study.

## 3. Discussion

### 3.1. The Genomic Variation Induced by Space Mutagenesis Has a Clear Genetic Tendency

Genomic variation is the foundation of biological genetic diversity and trait expression, and its ability to be stably inherited is influenced by various factors such as the type, location, function, and cell type of the variation [24,25,26]. In terms of mutation types, SNPs exhibit a stronger genetic tendency for intergenerational transmission compared to InDel [27]. The results of this study also demonstrate this phenomenon, with a significantly higher proportion of SNPs than InDel in the genomic variation loci in SP_1_-SP_2_. The location of mutations in the genome determines their functional importance and genetic predisposition. In model organisms such as humans and rice, most of the mutations associated with complex traits or diseases are not located in protein coding regions, but are enriched in noncoding regulatory regions (such as promoters, enhancers) or intergenic regions [28,29]. The variations in these regions contribute to the main part of trait heritability by regulating gene expression, showing a stronger genetic tendency. In contrast, although the number of nonsynonymous SNV that cause amino acid changes in the coding region is relatively small, they also have important genetic potential in certain traits due to their direct impact on protein function [25,30]. Although the genetic proportion of nonsynonymous SNV was also high in this study, synonymous SNV showed the same trend, and the proportion of variant inheritance in noncoding regulatory regions was lower than that in CDS regions. This result also reflects the specificity of space mutagenesis variation characteristics compared to natural variation. The genetic ability of variation is also related to the function of the gene it is located in. In the human genome, variations in functionally important and highly conserved positions are more likely to affect heritable diseases or phenotypes, and their variations are more likely to be inherited, while functional adaptive variations (such as environmental response) are not easily preserved [31]. In this study, the heritability of heritable loci was significantly associated with gene function. Variations in relatively conserved functional genes, including those involved in protein modification and basal metabolism, were more likely to be inherited. The heritability of a plant is directly determined by whether it is a somatic or germ cell in the cell lineage where variation occurs due to the phenomenon of cell chimerism [32,33]. The cell types in which variation exists affect the genetic efficiency of variation. In this study, the genetic rate of genomic variation in the samples ranged from 0 to 42.25%, which also reflected the influence of chimerism on the inheritance of variation. As the variation detection is based on somatic cells, the germ cells of low heritability samples may carry fewer variations, while high heritability samples carry more variations. In addition, there is a significant positive correlation between the heritability of sample variation and the number of sample variations. Therefore, increasing the number of variations in parents is also one of the methods to maintain a higher frequency of variation in offspring.

### 3.2. Genetic Patterns of DNA Methylation Changes

Environmental pressure or stress can induce DNA methylation changes, which can be inherited from offspring through sexual reproduction [34]. DNA methylation is a relatively stable epigenetic marker in plant sexual reproduction, which can be inherited by offspring, but most methylation marks are actively erased in parental gametes [35]. The results of this study confirmed this viewpoint, as the methylation sites of parents were not fully inherited by offspring, indicating that some methylation sites had been erased. Similar to genomic variation, the stability and tendency of DNA methylation inheritance are not uniform, mainly influenced by multiple factors such as methylation type, genomic location, and environmental factors. Methylation type is one of the core factors determining the heritability of DNA methylation. Among the three types of DNA methylation, CG methylation has a symmetrical structure, allowing DNA replication. Methyltransferases use the parent chain as a template to accurately replicate the methylation pattern to the daughter chain. This “maintenance methylation” mechanism ensures its high generation stability [36,37]. The CHG sequence also has symmetry, but its mechanism is not as stable as CG and is more susceptible to environmental or epigenetic drift [38]. CHH methylation, due to its asymmetric sequence, cannot be maintained through simple copy mechanisms and mainly relies on the “de novo methylation” pathway for re-establishment, resulting in significant dynamic changes and the weakest genetic predisposition [39]. The results of this study were consistent with the above conclusions. Among the heritable methylation sites, CG type had the highest proportion, followed by CHG, and CHH had the smallest proportion. Secondly, the genomic location determines the genetic potential of methylation patterns. Usually, methylation in repeat sequence regions such as transposable elements is more stable and conserved, with a higher genetic predisposition, while methylation in regions related to gene regulation is more dynamic and more susceptible to environmental factors, with a relatively lower genetic predisposition [40,41,42]. In this study, it was necessary to distinguish between DMC and DMR. The heritability of DMCs in gene regulation related regions was lower, while the heritability of coding regions was higher, while that of DMR was completely opposite. The coding region sequence had high conservation, and if the methylation of a single C site changes, it may affect codon function or mRNA stability. Therefore, evolution tends to stabilize inheritance through efficient maintenance mechanisms, resulting in a high heritability of DMC; however, the DMR in the coding region may lead to significant abnormalities in gene function, which are strictly limited by evolutionary selection and difficult to stably inherit, resulting in a low heritability. The methylation changes in a single C site in the regulatory region usually have limited and reversible effects on gene expression and are susceptible to environmental signal interference and cannot be stably inherited, resulting in a low heritability of DMC. However, the DMR in the regulatory region is a key unit for gene expression regulation, and its pattern is closely related to cell fate and environmental adaptation. It needs to be partially stably transmitted through specific epigenetic mechanisms, so the heritability is actually higher. Finally, environmental stress is also an important driving force for inducing heritable methylation changes. Stress such as drought and pathogen infection can trigger genome-wide methylation changes, some of which can evade “reprogramming” erasure during gamete formation and fertilization, and stably pass on to offspring, forming so-called “epigenetic mutations” [43,44]. The space environment in this study, as an extreme stress, can also serve as a powerful driving force to induce changes in DNA methylation throughout the rice genome. Some of the methylation mutations occur in key stress responsive gene regulatory regions, which can be stably transmitted to the next generation and form heritable “epigenetic mutations”. This mechanism provides an epigenetic basis for organisms to quickly adapt to environmental changes, which is of great significance in crop domestication and breeding.

### 3.3. The Potential Application of Candidate Genes in Rice Breeding

Space mutagenesis breeding can efficiently induce diverse mutations in the genome by exposing rice seeds to the space environment, providing abundant candidate gene resources for rice breeding. These candidate genes have shown significant potential in improving agronomic traits, enhancing stress tolerance, and accelerating breeding processes. Space mutagenesis can regulate key traits such as plant height and tillering, and the relevant candidate genes provide a molecular basis for cultivating dwarf or semi-dwarf lodging resistant varieties. The dwarf mutant genes identified by radiation mutagenesis can be stably inherited and directly used for high-yield breeding [45]. The cloning of genes such as *OsSAUR11* laid the foundation for improving root systems, enhancing drought tolerance, and maintaining yield [46]. Space mutagenesis candidate genes can help cultivate cold resistant, heat resistant, and hypoxia tolerant varieties. The cold stress-related genes identified through genome-wide association analysis (GWAS) can be used to optimize germination and growth under low temperature conditions; high temperature responsive genes help maintain the source sink balance during the grain filling period and reduce yield losses [47,48]. In addition, the ROS response pathway and MAPK cascade genes activated by space mutagenesis provide new strategies for rice to cope with extreme environments [49]. Space mutagenesis breeding is closely integrated with modern biotechnology, significantly improving breeding efficiency. Technologies such as high-throughput sequencing, RNA-seq, and GWAS have accelerated the identification and functional verification of candidate genes. Molecular marker assisted selection (MAS) and CRISPR gene editing have achieved precise aggregation and targeted improvement in key genes, shortening the breeding cycle [50,51,52]. This study utilized a space-induced multi-trait mutant library combined with RNA-seq screening to identify seven candidate genes, demonstrating enormous potential for breeding applications in improving nutrient efficiency, enhancing environmental tolerance, and optimizing yield composition. For nitrogen utilization, *OsPho1* (*SK1G00044581*) belongs to the rice *PHO1* gene family, with *OsPHO1;2* playing a key role in root-to-shoot phosphate transport [53]. Although *OsPho1* is primarily associated with phosphate homeostasis, phosphate and nitrogen metabolism are functionally interconnected, and mutations affecting phosphate transport may indirectly influence nitrogen utilization efficiency. *CRK25* (*SK1G00052825*) encodes a cysteine-rich receptor-like kinase, a class of proteins known to participate in stress signal perception and transduction. Receptor-like kinases often function in nutrient signaling pathways [54]; thus, the frameshift mutation in *CRK25* may disrupt nitrogen-related signal transduction, contributing to the chlorate insensitive phenotype observed in the K125 mutant. For drought tolerance, the three candidate genes have well-established roles in stress responses. *PER1* (*SK1G00042488*) encodes a 1-cysteine peroxiredoxin, a member of the antioxidant enzyme family that scavenges reactive oxygen species under stress conditions. Peroxidase family members play important roles in coping with drought stress by removing reactive oxygen species, and *PER1* expression is associated with stress and oxidative stress responses [55]. *RCN1* (*SK1G00048986*) is a TERMINAL FLOWER-like gene that enhances drought tolerance by promoting ABA biosynthesis and facilitating new root germination. It also contributes to stomatal closure through ABA accumulation in guard cells and helps maintain spike structure under drought stress by promoting secondary branch formation [56]. *OsPUP1* (*SK1G00051356*) encodes a purine permease that modulates cytokinin distribution. Given that cytokinins are key regulators of shoot/root balance and stress responses, the frameshift mutation in *OsPUP1* may alter cytokinin homeostasis, thereby affecting drought response mechanisms [57]. For cold tolerance, *cys12* (*SK1G00061753*) corresponds to the CTS-12 locus derived from wild rice, which mediates chilling tolerance through ABA-dependent stomatal opening modulation. The CTS-12-mediated ABA signaling pathway triggers transcriptional regulation of chilling-responsive genes and metabolic shifts that coordinately regulate guard cell stomatal movement under chilling stress [58]. *OsRP1L1* (*SK1G00048468*) encodes an NB-LRR (nucleotide binding site–leucine-rich repeat) protein. While initially characterized by its role in bacterial blight resistance, *OsRP1L1* expression is also suppressed by high salt, high temperature, and high osmotic stress, and it is considered to participate in responses to multiple abiotic stresses [59]. The frameshift mutation in *OsRP1L1* may thus compromise its function in stress signal transduction, contributing to the cold-tolerant phenotype. It should be noted that plant drought and cold tolerance are complex quantitative traits governed by multiple genes and pathways, and the seven genes identified here represent only a subset of the genetic determinants underlying these traits. Nonetheless, the consistent co-segregation of frameshift mutations in these genes with the target phenotypes, combined with their known or predicted functions in stress-related and nutrient-related pathways, strongly supports their candidacy as key regulators.

### 3.4. Research Limitations

This study has several limitations. First, chlorate insensitivity is traditionally associated with reduced nitrate uptake or decreased nitrate reductase (NR) activity, rather than directly reflecting enhanced nitrogen use efficiency (NUE). Although the mutants identified in this study exhibited significantly higher biomass than the wild type under nitrogen-limited conditions with nitrate as the sole nitrogen source, we did not directly measure nitrate uptake rates, NR activity, or total nitrogen accumulation. Therefore, the interpretation of these mutants as having improved NUE remains preliminary and requires further physiological validation. Second, the drought-tolerant mutants identified in this study were primarily screened based on germination performance under PEG-6000-simulated osmotic stress. However, the relationship between osmotic tolerance at the germination stage and actual drought resistance under field conditions is not straightforward. Although the candidate genes and mutants obtained in this study provide resources for further functional studies, their practical value for improving field drought resistance needs to be systematically validated across multiple environments and different growth stages. Third, although the mutants were identified using a germination-based screening strategy, the genetic and physiological mechanisms underlying cold tolerance at germination may differ from those at the vegetative or reproductive stages; therefore, their cold tolerance at later growth stages requires further verification. Finally, it should be noted that this study was based on the Chang’e-5 spaceflight mission, and the experimental design did not allow individual space environmental factors, such as cosmic radiation and microgravity, to be isolated as independent mutagenic drivers. Rice seeds were exposed to the integrated deep-space environment during the mission, and no ground-based controls capable of isolating individual factors were included. This limitation is inherent to space biology studies of an opportunistic mission nature.

## 4. Materials and Methods

### 4.1. Chang’e-5 Carried Seeds and Materials for Planting

The material carried on Chang’e-5 this time is pure line HJXSM of indica rice, which was selected by the National Plant Aerospace Breeding Engineering Technology Research Center of South China Agricultural University (SCAU) through hybridization of disease resistant and high-quality indica rice variety Huahang 31 and Hanghui 1508 using the pedigree method. This strain aggregates multiple disease tolerant genes and aroma genes, and has stable agronomic traits. On 24 November 2020, dried HJXSM weighing approximately 32.8 g from the same individual plant was loaded onto Chang’e-5 lunar probe, with a total flight time of approximately 23 days.

After returning to Earth, the HJXSM seeds carried by Chang’e-5 were sown on 20 February 2021, at the Teaching and Research Base of SCAU. The seeds were soaked at 30 °C for 48 h and pregerminated at 35 °C for 24 h, and then sown in a nursery bed using the wet nursery method. Then, 30-day-old seedlings were transplanted to the field. A total of 2500 plants were grown in the SP_1_ generation, with one seedling planted per hill and both row and plant spacing set at 20 cm. At maturity, each SP_1_ plant was harvested and threshed individually. From each plant with normal seed setting, 200 seeds were harvested, whereas all seeds were harvested from a few plants with weak growth vigor or affected fertility. All harvested seeds were stored under individual plant numbers for subsequent planting of SP_2_ lines. The SP_2_ generation was planted using a line-plot design. The progeny of each SP_1_ plant constituted one line, giving a total of 2500 lines. Ten plants were planted per line in sequential order, with plant and row spacing consistent with those in the SP_1_ generation.

### 4.2. Sampling of WGS and WGBS Materials

A total of 30 SP_1_ plants (TK1-TK30) and 10 WT plants (CK1-CK10) at the jointing stage were randomly selected, and one young stem was collected from each tiller for subsequent research. A total of 30 TK group materials were bagged and self-pollinated, and seeds were harvested and planted into SP_2_ lines (each line containing 10 individual plants). Samples were taken from 30 SP_2_ lines, and one tender stem from a tiller was collected from each material. The tender stems of 10 individual plants from each line were mixed and renumbered as NTK1-NTK30.

### 4.3. DNA and RNA Extraction and Quality Inspection

Approximately 500 mg of each sample was used. DNA was extracted using the cetyltrimethylammonium bromide (CTAB) method, total RNA was extracted using the Omega Plant RNA kit (Omega Bio Tek, GA, USA, R6827), and DNA and RNA mass were detected using Qubit (Thermo Fisher Scientific, MA, USA) and Nanodrop (Thermo Fisher Scientific, USA). The integrity of RNA was determined using Agilent 2100 (Agilent Technologies, Germany). Samples that passed quality inspection were stored at −80 °C.

### 4.4. WGS and Authentic Variant Site Screening

DNA samples that passed quality inspection were sequenced on Illumina machines. Firstly, quality control was performed using Fastp (v0.20.0) on Illumina sequencing data. Then, BWA (v0.7.15) was used to align the filtered reads to the reference genome SK1, which serves as the reference genome for the background of HJXSM constructed earlier. GATK (v3.4-46) was used for mutation detection and ANNOVAR (v2) was used for functional annotation to obtain the original mutation data. To screen the original mutation data, first, loci with reads < 5 were removed. Among the 30 TK samples, variant sites present in ≥2 TK samples were removed (considered as population background polymorphisms prior to space mutagenesis); similarly, among the 30 NTK samples, variant sites present in ≥2 NTK samples were removed (considered as common background loci in the SP_2_ population). The “TK true variants” obtained after within-group filtering of the TK group and the “NTK true variants” obtained after within-group filtering of the NTK group were subjected to intersection analysis sites present in both the TK parent and its corresponding NTK progeny were defined as “inherited loci.” Variant sites in the CK samples were used to assist in determining which sites represent the fixed genetic background of the line itself, and were not involved in cross-sample filtering or exclusion.

### 4.5. WGBS and Analysis

A DNA library for bisulfite sequencing was prepared using DNA that had passed quality inspection, including 10 CK, 30 TK, and 30 NTK samples. The specific steps were as follows: genomic DNA was sonicated (Covaris, MA, USA) and fragmented into 100–300 bp fragments, which were then purified using the MiniElute PCR Purification Kit (QIAGEN, USA). Subsequently, these fragments underwent terminal repair and an ‘A’ nucleotide was added at the 3′ end. Afterwards, the genome fragments were connected to methylation sequencing adapters. The fragment with a linker was subjected to bisulfite conversion using the Methylation Gold Assay Kit (ZYMO, CA, USA). During the sodium bisulfite treatment, unmethylated cytosine was converted to uracil. Finally, the transformed DNA fragments were amplified by PCR and subjected to Illumina sequencing. The raw sequencing data was converted into sequence data raw data through base calling, and the filtered data was aligned to the genome sequence using BSMAP (v2.90) to obtain all genome base alignment information and all genome C-base methylation information. DMCs were outputted using MethylKit (v1.4.1). The entire genome was scanned with a 200 bp window, the average DNA methylation rate was calculated within each window, the methylation levels of each sample were compared within each window, and DMRs were outputted.

### 4.6. Screening of Nitrogen Utilization Mutants

In total, 100 clean and plump seeds were selected from each SP_2_ material, washed, disinfected, soaked in sterile water, and sown into PCR plates with holes at the bottom after radicle emergence. First, we cultivated them in sterile water for 2 days, and then replaced them with improved nitrogen deficient Hoagland nutrient solution (formula standard dose: 1.678 g/L). Element content: NH_4_^+^ 0 mmol, NO_3_^−^ 0 mmol, P 1 mmol, K 6 mmol, Ca 4 mmol, Mg 2 mmol, S 4.5 mmol, Cl 8 mmol, and add 2 mmol/L KNO_3_ as the sole nitrogen source. The cultivation temperature was 30 °C, the relative humidity was 70%, and the light cycle was 14 h of light and 10 h of darkness. After 4 days, it was replaced with modified Hoagland nutrient solution containing different concentration gradients of KClO_3_ (1 mmol/L, 2 mmol/L, and 3 mmol/L) and cultured for another 4 days. Each treatment was repeated three times. After cultivation, the entire seedling was scanned using a scanner and Canon MP Navigator (v4.0.9), and its phenotypic characteristics were recorded. Seedling length was measured and chlorate sensitivity was calculated using Image Pro Plus (v6.0), allowing for the preliminary screening of nitrogen utilization mutants. Using the same method described above, the mutant lines preliminarily screened in SP_2_ were subjected to chlorate sensitivity testing again in SP_3_, resulting in the final identification of nitrogen utilization mutants.

Chlorate sensitivity = (WT plant height − mutant plant height)/WT plant height × 100%

### 4.7. Screening of PEG-Induced Tolerant Mutants

From each SP_2_ material, 100 clean and plump seeds were selected, cleaned, disinfected, and subjected to PEG-induced treatment with a 22% PEG-6000 solution. Three replicates were set up per material and cultured under the aforementioned conditions. Germination was defined as an embryo bud length of ≥0.5 mm. The number of germinated seeds was recorded on the 7th day, the germination rate was calculated, and a preliminary screening for PEG-induced tolerant mutants was conducted. For the candidate PEG-induced resistant materials preliminarily screened from SP_2_, PEG-induced treatment was repeated at the SP_3_ stage, and the germination rate was assessed again.

Germination rate = (number of germinated seeds in the 7th day/total number of tested seeds) × 100%

### 4.8. Screening of Germination-Stage Cold Tolerant Mutants

For each SP_2_ material, 100 clean and plump seeds were selected, cleaned, disinfected, and cultured in sterile water at 15 °C. Three replicates were maintained per material under otherwise standard conditions. The germination rate was calculated every 24 h over 10 consecutive days. Based on this data, the average germination days and germination coefficient were calculated to conduct a preliminary screening for germination-stage cold-tolerant mutants. To verify the phenotype, the candidate germination-stage cold-tolerant materials identified in SP_2_ were subjected to germination-stage cold stress treatment again in SP_3_, and their germination coefficient was determined.

Average germination days = (∑ number of germinated grains on the day × number of days after soaking)/total number of grains in the experiment

Germination coefficient = germination rate/average germination days

### 4.9. RNA-Seq

Seeds representing mutant SP_3_ were taken, each treatment was repeated three times, and the corresponding WT was set. K125 was sampled on the 8th day (K125-1), the 12th day KNO_3_ control group (K125-2), and the 12th day KClO_3_ treatment group (K125-3) of the seedling stage. K12 was taken on the 3rd day (K12-1), the 5th day (K12-2), and the 7th day (K12-3) of seed germination under 22% PEG-6000 solution treatment. K44 was taken on the 6th day of seed germination under 15 °C cold stress treatment.

After extracting high-quality total RNA from the processed samples, library construction and Illumina sequencing were performed. Comparative analysis was conducted based on reference genome using HISAT (v2.1.0), including type statistics, gene coverage, sequencing randomness, and sequencing saturation analysis. Transcripts were reconstructed using Stringtie (v2.2.3) and RSEM (v1.3.3) was utilized to calculate the expression levels of all genes in each sample, display them as fragments per kill of exon model per million mapped fragments (FPKM), and use FPKM as a screening criterion. DEGs were output with |log2FC| ≥ 1 and FDR < 0.05. The target gene was transferred to the GO database (http://www.geneontology.org/) to map each term to obtain GO terms with significantly enriched genes. The target gene set was integrated with the KEGG database (https://www.kegg.jp/) to combine enrichment analysis to screen for significantly enriched pathways.

### 4.10. qRT-PCR

Total RNA was extracted from samples of the WT and the aforementioned treatments. mRNA was reverse transcribed using the Evo M-MLV Reverse Transcription Kit (AGbio, CO, AG11728). qRT-PCR was performed on a CFX96 instrument (Bio-Rad, USA). The PCR reaction was conducted using target gene-specific primers, with UBQ (*Os03g13170*) serving as the internal reference gene. The relative expression level of the target gene under each treatment was determined by calculating the 2^−ΔCt^ value, with three biological replicates included for each assay. The sampling time point was consistent with the time point at which the RNA-seq differences in the target genes were most significant. The primer sequences are provided in Table S1.

### 4.11. Statistical Analysis

In the statistical analysis, data was presented as the mean ± standard deviation, with error bars included. A two-tailed *t*-test was used to determine significant differences between two groups. For datasets containing three or more experimental groups, one-way ANOVA was performed using IBM SPSS software (v21.0), followed by Duncan’s multiple range test. Differences with a *p*-value less than 0.05 were considered statistically significant (* *p* < 0.05, ** *p* < 0.01, *** *p* < 0.001).

## 5. Conclusions

This study systematically reveals the transgenerational inheritance patterns of genomic and DNA methylation variations induced by the Chang’e-5 deep-space environment in rice. The results demonstrate that the number of genomic variants in the SP_2_ generation increased significantly compared with SP_1_, and that SNPs, homozygous sites, and variants in coding regions (CDS) were more stably inherited. The genome-wide methylation level was elevated in the SP_2_ generation, and among differentially methylated sites, those in the CG context exhibited the highest heritability. Through large-scale phenotypic screening, a collection of stably inherited mutants with high nitrogen efficiency, PEG-induced tolerance, and germination-stage cold tolerance was obtained. By integrating whole-genome variant analysis and multi-time-point transcriptomic profiling of representative mutants, seven candidate genes regulating the target traits were identified. This study deepens the theoretical understanding of deep-space mutagenesis mechanisms and provides valuable germplasm resources and functional gene targets for precision space breeding in rice.

## Figures and Tables

**Table 1:** Information on 7 candidate genes.

SK1 ID	IRGSP-1.0 ID	Gene Name	Mutation Location	Functional Mutation Type	Amino Acid Changes	Related Traits
*SK1G00044581*	*Os03g0758100*	*OsPho1*	Chr3_5569159	Frameshift deletion	K778fs	Nitrogen utilization
*SK1G00052825*	*Os04g0322100*	*CRK25*	Chr4_16033435	Frameshift deletion	N159fs	Nitrogen utilization
*SK1G00042488*	*Os01g0207900*	*PER1*	Chr1_30792148	Frameshift insertion	A227fs	PEG-induced tolerant
*SK1G00048986*	*Os03g0281900*	*RCN1*	Chr3_7476262	Frameshift insertion	Q23fs	PEG-induced tolerant
*SK1G00051356*	*Os03g0187800*	*OsPUP1*	Chr3_34595972	Frameshift deletion	L7fs	PEG-induced tolerant
*SK1G00061753*	*Os03g0215800*	*cys12*	Chr3_7620043	Frameshift insertion	E32fs	Germination-stage cold-tolerant
*SK1G00048468*	*Os05g0365300*	*OsRP1L1*	Chr5_3361761	Frameshift insertion	I141fs	Germination-stage cold-tolerant

## Data Availability

All data in this study can be found in the manuscript or the Supplementary Materials.

## Supplementary Materials

The following supporting information can be downloaded at: https://www.mdpi.com/article/10.3390/plants15172614/s1; Figure S1: Distribution of TK and NTK genomic variation sites on chromosomes, where the color represents the number of mutation sites; Figure S2: Comparison of genomic variation characteristics and number of heritable loci between TK and NTK; Figure S3: Enrichment analysis of genes containing heritable loci and genes containing non-heritable loci; Figure S4: Comparison of methylation rates of TK and NTK methylation types on the genome; Figure S5: Distribution of DMCs and DMRs on chromosomes; Figure S6: Representative mutants; Figure S7: Enrichment analysis of DEGs with different treatments at K125; Figure S8: Enrichment analysis of DEGs with different treatments at K12; Figure S9: DEGs enrichment analysis of K44; Figure S10: Heatmap of expression levels of marker genes related to nitrogen utilization, drought stress, and cold stress; Table S1: qRT-PCR Primer Information.
