# Supplementary material for: Genetic Analysis of Genomic and Methylomic Variation and Identification of Multi-Trait Mutants in Rice Carried on Chang’e-5
Source: Plants (Basel). 2026 Aug 27;15(17):2614. doi: 10.3390/plants15172614 (PMC13567658; doi:10.3390/plants15172614)
Supplement: Supplementary file 1 [file plants-15-02614-s001.zip › Supplementary Figures.pdf]

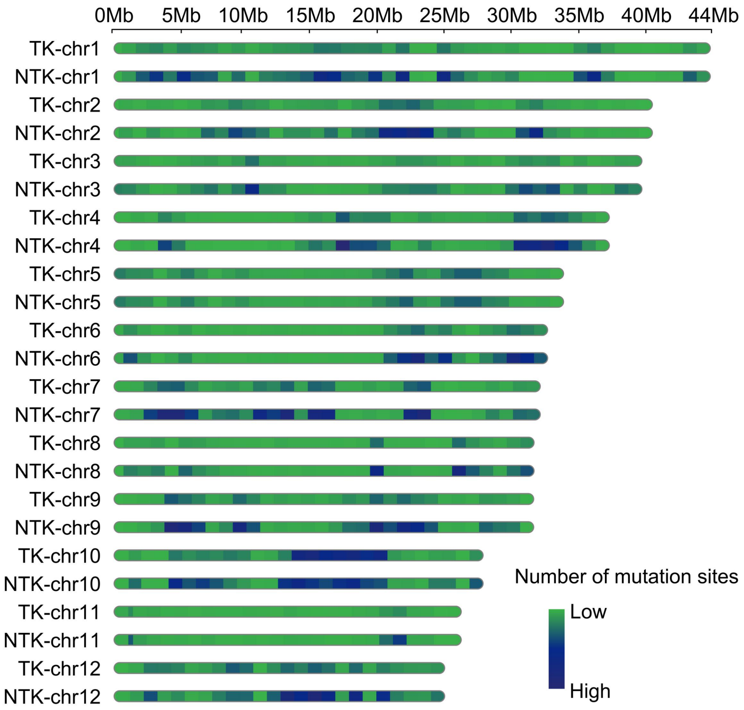

Figure S1. Distribution of TK and NTK genomic variation sites on chromosomes, where the colour represents the number of mutation sites.

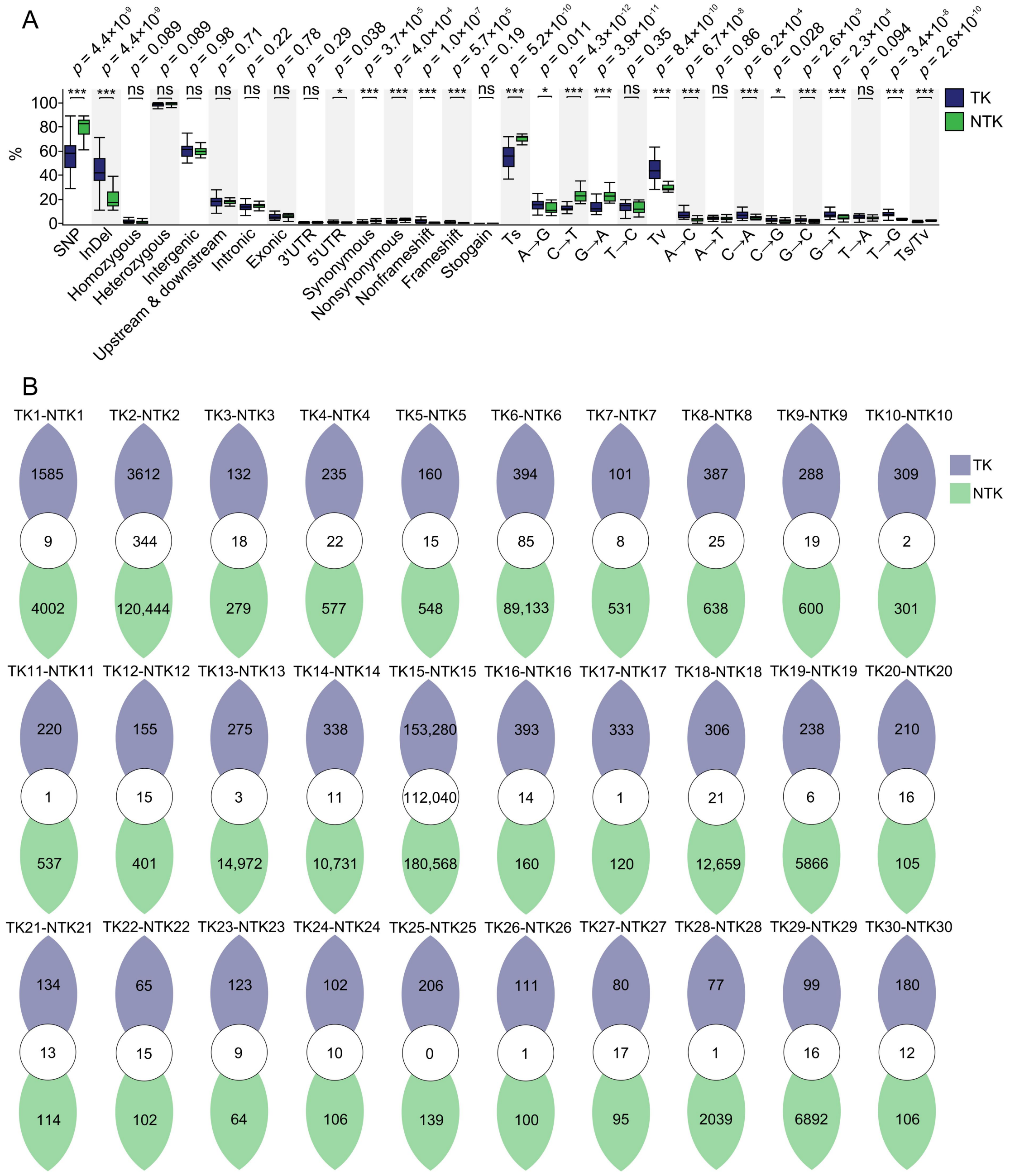

Figure S2. Comparison of genomic variation characteristics and number of heritable loci between TK and NTK. (A) Comparison of genomic variation characteristics between TK and NTK. (B) The number of heritable and non-heritable variant loci in TK and NTK single group samples.

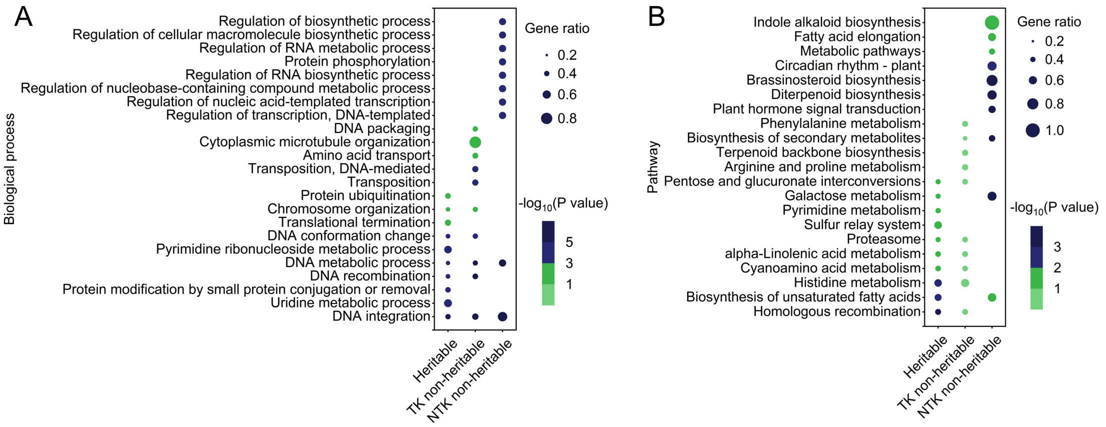

Figure S3. Enrichment analysis of genes containing heritable loci and genes containing non-heritable loci. (A) GO enrichment with heritable loci and non-heritable loci, selecting the top 10 biological processes with the highest significance, where the circle size represents the ratio of target genes enriched in the biological process to background genes enriched in the biological process, and the colour represents the significance level. (B) Enrichment of KEGG genes containing heritable loci and genes containing non-heritable loci, selecting the top 10 pathways with the highest significance, where the circle size represents the ratio of target genes enriched in the pathway to background genes enriched in the pathway, and the colour represents the significance level.

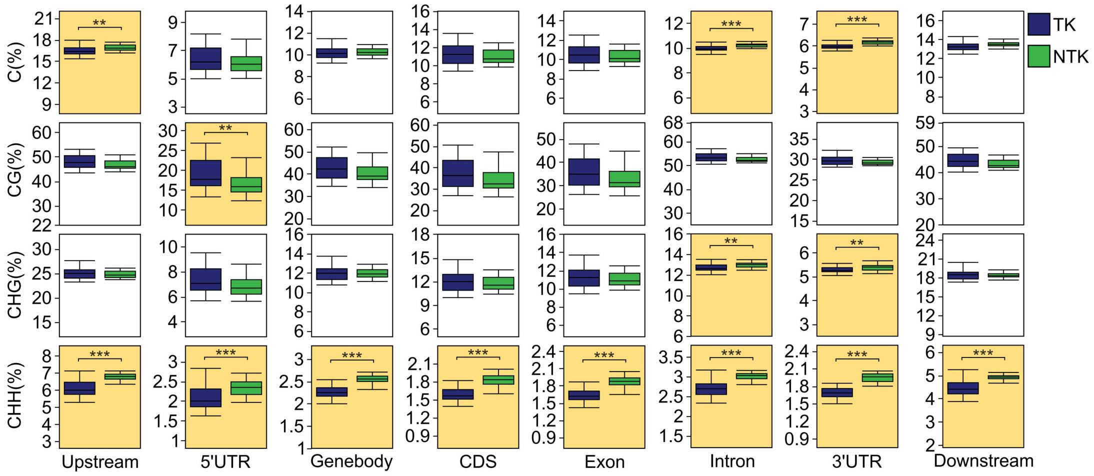

Figure S4. Comparison of methylation rates of TK and NTK methylation types on the genome.

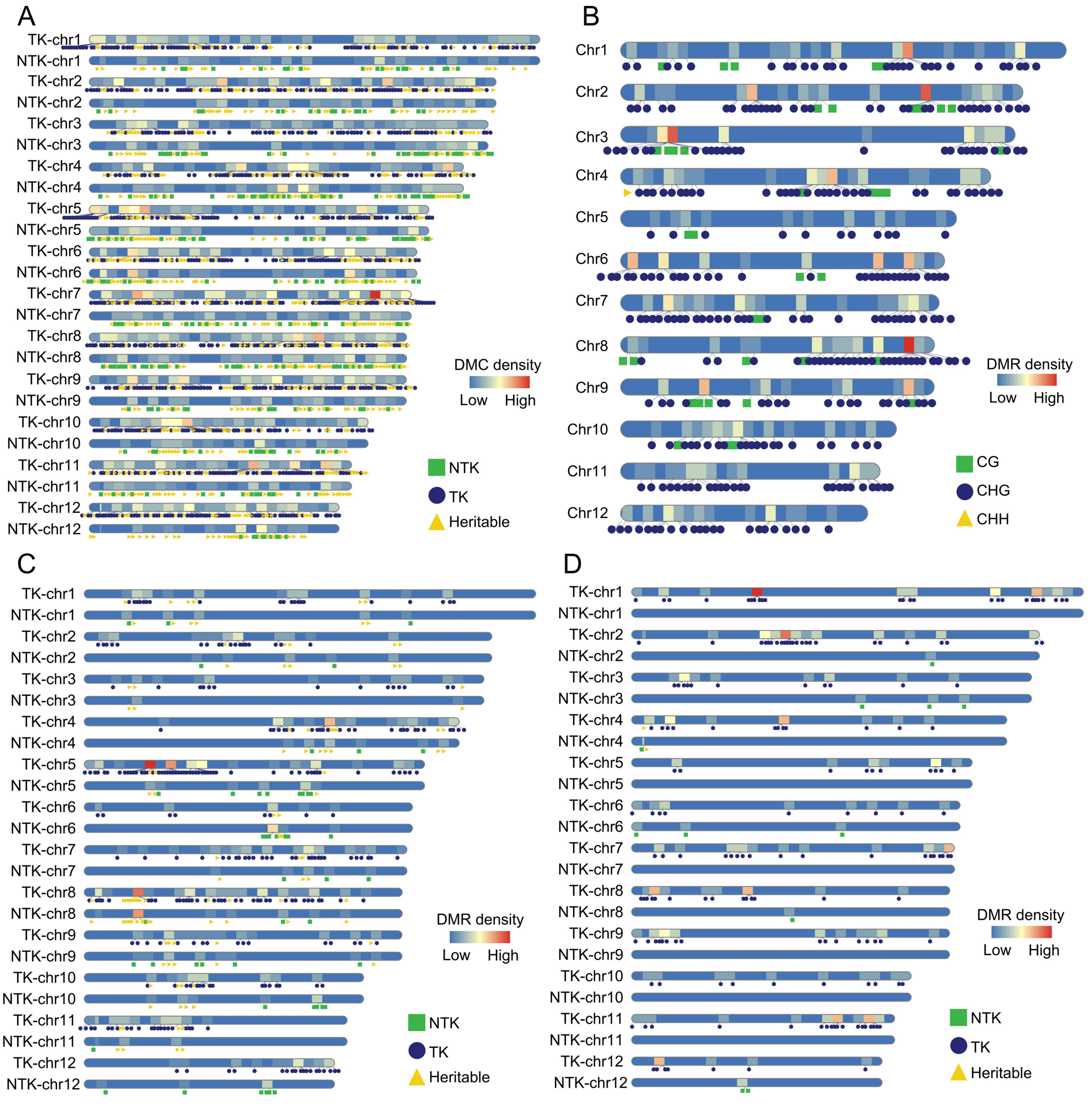

Figure S5. Distribution of DMCs and DMRs on chromosomes. (A) The distribution of heritable and non-heritable DMCs on chromosomes, where the colour represents the density of DMC. (B) The distribution of three types of DMRs on chromosomes, where the colour represents the density of DMR. (C) The distribution of heritable and non-heritable DMRs of CG type on chromosomes, where the colour represents the density of DMR. (D) The distribution of heritable and non-heritable DMRs of CHG type on chromosomes, where the colour represents the density of DMR.

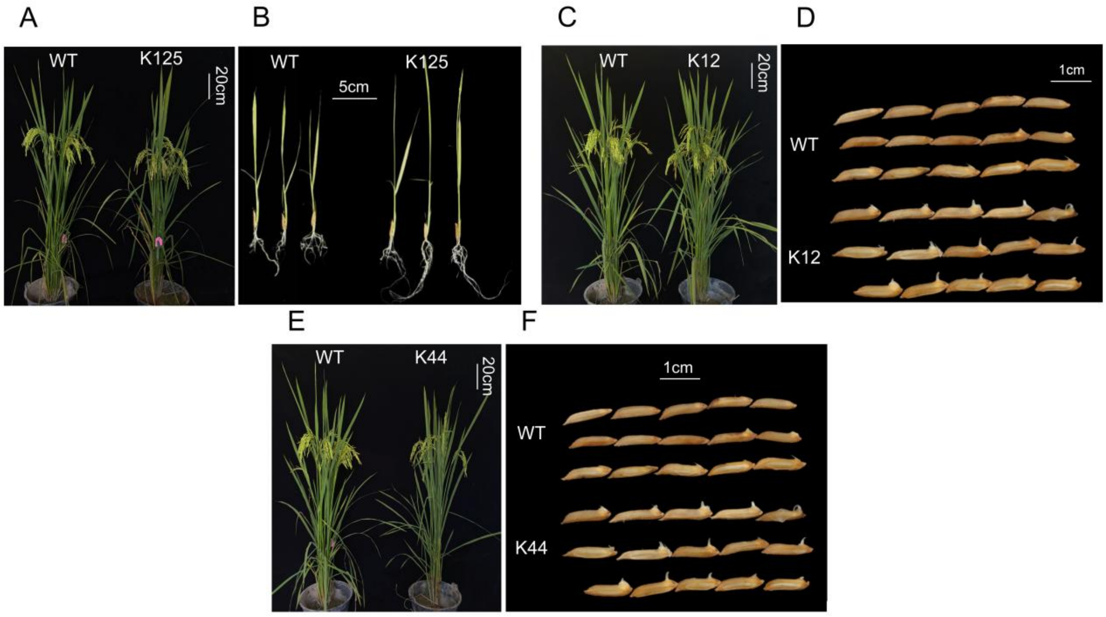

Figure S6. Representative mutants. (A) Nitrogen-efficient mutant K125. The vertical bar represents 20 cm. (B) Comparison of chlorate sensitivity between K125 and WT. The vertical bar represents 5 cm. (C) PEG-induced tolerant mutant K12. The vertical bar represents 20 cm. (D) Comparison of germination rates of K12 and WT under PEG-induced treatment. The vertical bar represents 1 cm. (E) Germination-stage coldtolerant mutant K44. The vertical bar represents 20 cm. (F) Comparison of germination rates between K44 and WT under cold treatment. The vertical bar represents 1 cm.

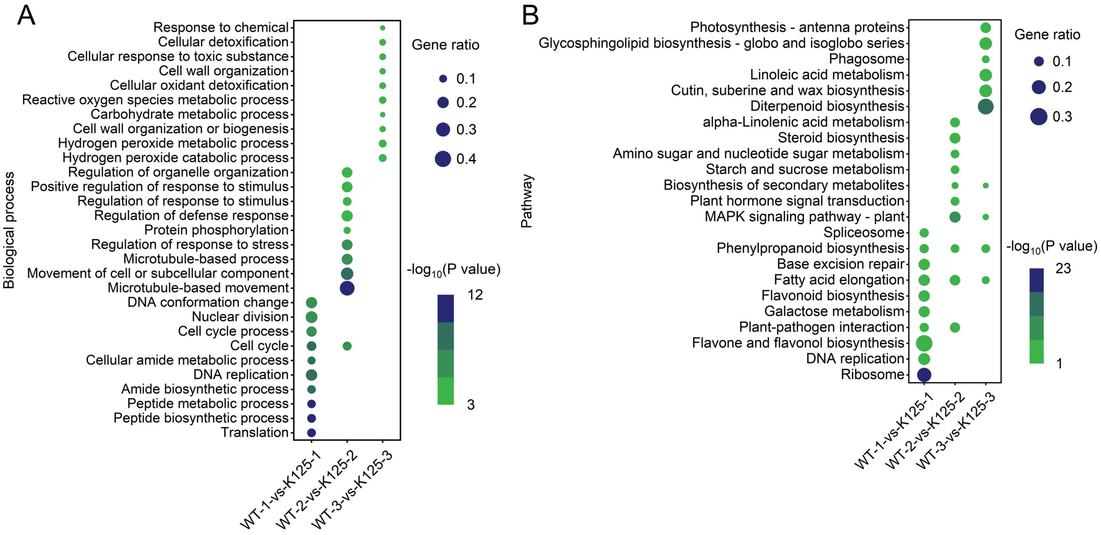

Figure S7. Enrichment analysis of DEGs with different treatments at K125. (A) GO enrichment of DEGs in different treatments, selecting the top 10 biological processes with significant differences, where the circle size represents the ratio of target genes enriched in the biological process to background genes enriched in the biological process, and the colour represents the significance level. (B) KEGG enrichment of DEGs in different treatments, selecting the top 10 pathways with significant differences, where the circle size represents the ratio of target genes enriched in the pathway to background genes enriched in the pathway, and the colour represents the significance level.

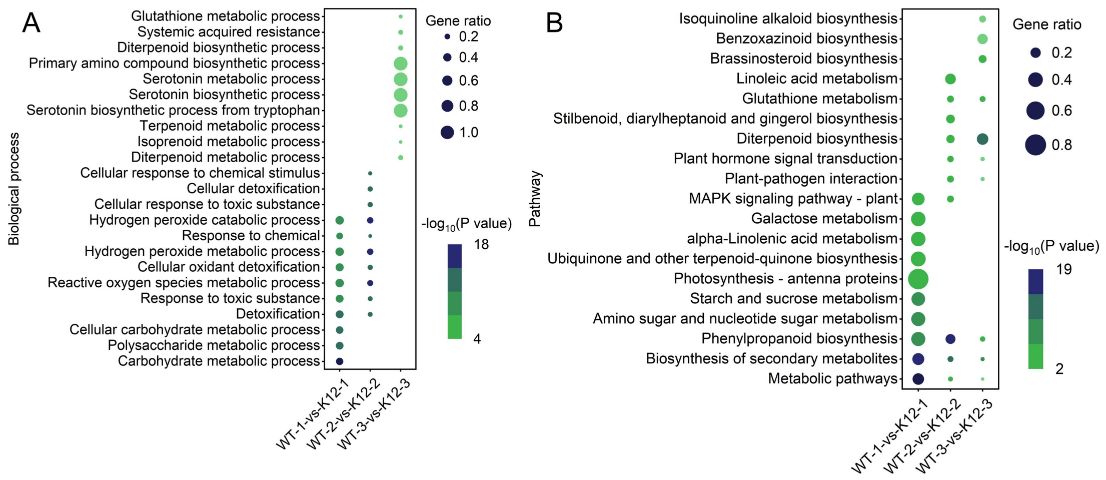

Figure S8. Enrichment analysis of DEGs with different treatments at K12. (A) GO enrichment of DEGs in different treatments, selecting the top 10 biological processes with significant differences, where the circle size represents the ratio of target genes enriched in the biological process to background genes enriched in the biological process, and the colour represents the significance level. (B) KEGG enrichment of DEGs in different treatments, selecting the top 10 pathways with significant differences, where the circle size represents the ratio of target genes enriched in the pathway to background genes enriched in the pathway, and the colour represents the significance level.

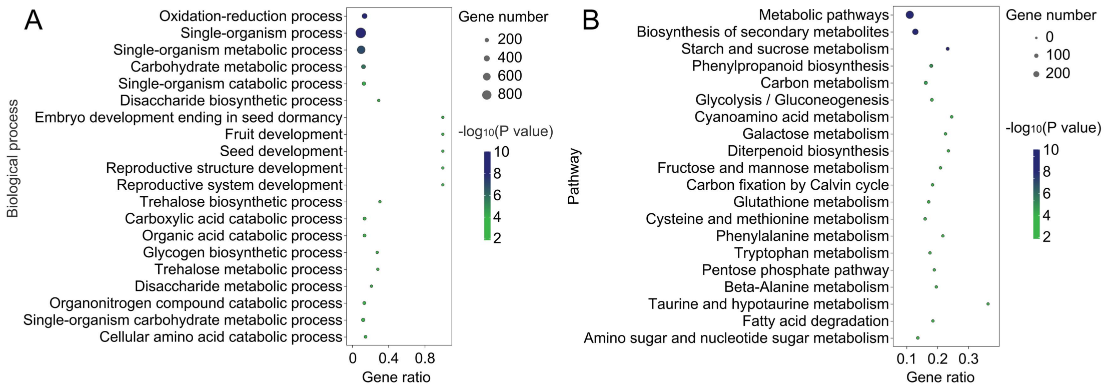

Figure S9. DEGs enrichment analysis of K44. (A) GO enrichment of DEGs in different treatments, selecting the top 10 biological processes with significant differences, where the circle size represents the number of target genes enriched in the biological process, and the colour represents the significance level. (B) KEGG enrichment of DEGs in different treatments, selecting the top 10 pathways with significant differences, where the circle size represents the number of target genes enriched in the pathway, and the colour represents the significance level.

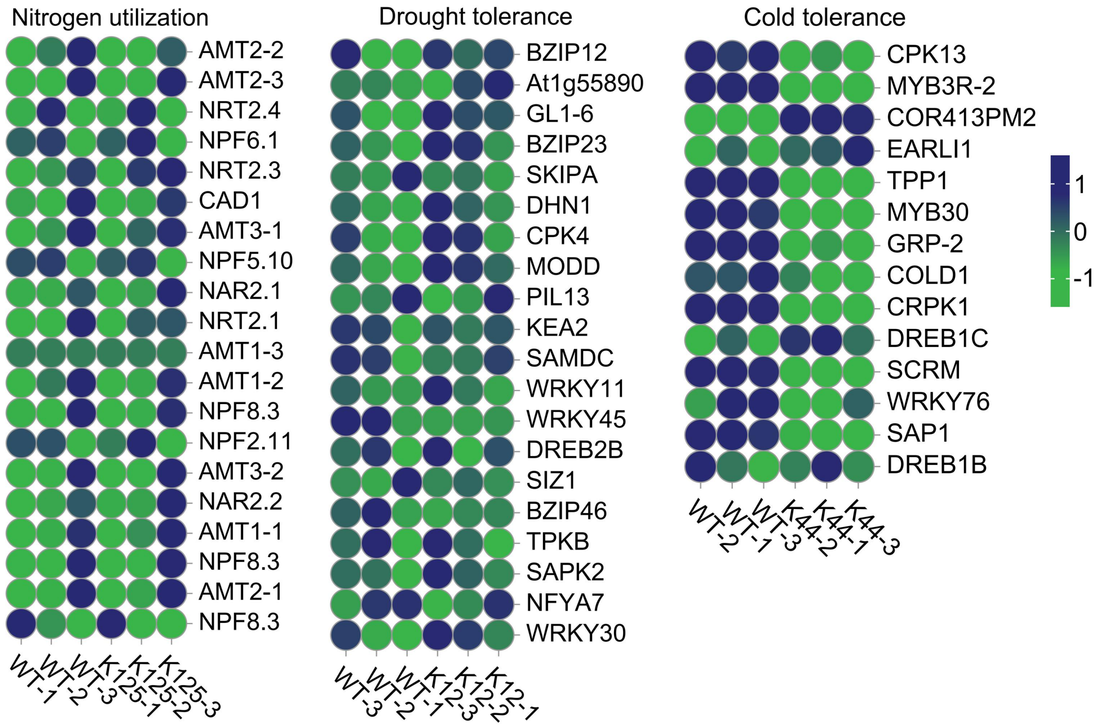

Figure S10. Heatmap of expression levels of marker genes related to nitrogen utilization, drought stress, and cold stress.
